# Ethics of Digital Mental Health During COVID-19: Crisis and Opportunities

**DOI:** 10.2196/23776

**Published:** 2020-12-22

**Authors:** Nicole Martinez-Martin, Ishan Dasgupta, Adrian Carter, Jennifer A Chandler, Philipp Kellmeyer, Karola Kreitmair, Anthony Weiss, Laura Y Cabrera

**Affiliations:** 1 Department of Pediatrics, Center for Biomedical Ethics, School of Medicine Stanford University Stanford, CA United States; 2 Department of Philosophy University of Washington Seattle, WA United States; 3 School of Psychological Sciences and the Turner Institute for Brain and Mental Health Monash University Melbourne Australia; 4 Faculty of Law, Centre for Health Law, Policy & Ethics University of Ottawa Ottawa, ON Canada; 5 Neuroethics and AI Ethics Lab Department of Neurosurgery University Medical Center Freiburg Freiburg Germany; 6 Department of Medical History and Bioethics, School of Medicine and Public Health University of Wisconsin Madison, WI United States; 7 Department of Psychiatry and Center for Bioethics Harvard Medical School Boston, MA United States; 8 Center for Ethics & Humanities in the Life Sciences, Department of Translational Neuroscience Michigan State University East Lansing, MI United States

**Keywords:** ethics, digital mental health, neuroethics, mental health, COVID-19, crisis, opportunity, implementation, online tool, telehealth

## Abstract

Social distancing measures due to the COVID-19 pandemic have accelerated the adoption and implementation of digital mental health tools. Psychiatry and therapy sessions are being conducted via videoconferencing platforms, and the use of digital mental health tools for monitoring and treatment has grown. This rapid shift to telehealth during the pandemic has given added urgency to the ethical challenges presented by digital mental health tools. Regulatory standards have been relaxed to allow this shift to socially distanced mental health care. It is imperative to ensure that the implementation of digital mental health tools, especially in the context of this crisis, is guided by ethical principles and abides by professional codes of conduct. This paper examines key areas for an ethical path forward in this digital mental health revolution: privacy and data protection, safety and accountability, and access and fairness.

## Introduction

COVID-19 is presenting a mental health crisis of unprecedented scale, due to the social and psychological burdens stemming from the pandemic, including social isolation, widespread unemployment, worries over contracting the virus, insomnia, social media exposure, and the rising death toll [1,2]. Mental health studies conducted during the pandemic have confirmed that symptoms of acute stress, anxiety, and depression, as well as suicidality, have been increasing [3-5]. Pre-existing mental health and health issues, secondary stressors such as job loss, and greater exposure to pandemic-related media coverage are the factors most strongly associated with increases in depressive and acute stress symptoms [6]. Social distancing measures also contribute to cognitive decline, substance abuse, and other mental health problems [7,8]. As is often the case, these negative effects disproportionately affect vulnerable groups in society, such as older adults, racial and ethnic minorities, people living with disabilities, people who are neurologically atypical, children, and people who are homeless [9-11]. People whose illness is serious enough to require admission to an intensive care unit may experience lingering trauma [12,13], and health care workers are experiencing extreme distress [14].

To address this mental health crisis and provide physically distanced care, there has been a major, accelerated move toward adoption and implementation of digital tools for mental health care [15-17]. Psychiatry and therapy sessions are being conducted via videoconferencing platforms, and digital tools for diagnosis, monitoring, and treatment, such as mental health apps, are increasing in use [18]. As the pandemic continues, telehealth is increasingly becoming the “new normal” in mental health care. Many in the mental health field have noted the upside to the increased use of digital mental health tools, such as broader access and lower rates of “no-shows” to appointments. Yet, given that privacy and safety regulations in the United States were relaxed to facilitate this new stage of mental health care [19] and the large scale in which these tools are being used, there is renewed urgency to assess and address ethical issues presented by digital mental health tools, such as lack of evidence of efficacy; privacy and data protection; access; and fairness, transparency, and accountability [20-22].

The urgent need for socially distanced mental health care should not be used to erode the regulations and practices that protect people from substandard mental health care or unwanted uses of their personal information. During this public health emergency, the need to protect people from infection by increasing tools for socially distanced care could certainly be seen as having greater weight than protecting privacy or cautious oversight regarding safety or effectiveness. Nevertheless, it remains important to examine the burdens, as well as benefits, and assess the appropriateness of the trade-offs being made to permit expanded digital mental health care. This paper examines key areas for addressing ethical digital mental health care in the wake of COVID-19: privacy and data protection, safety and accountability, and access and fairness.

## Privacy and Data Protection

Although privacy and data protection are important ethical considerations for health technologies, in digital mental health technologies, they become major concerns due to the sensitive personal information people share through digital mental health tools, as well as the potential for government and corporate misuse and surveillance. Mental health data is considered more sensitive than other health data [23] and can often be more personal or stigmatizing in nature. In the United States, to facilitate the provision of telehealth, the Office of Civil Rights at the Department of Health and Human Services issued a notification on March 30, 2020, altering the Health Information Portability and Accountability Act (HIPAA) Privacy Rule to eliminate penalties for health care providers for violations during the good faith provision of telehealth [24]. This rule change is limited to the provision of telehealth through non–public-facing communication products such as Zoom (Zoom Video Communications, Inc) [25]. Privacy issues that arose in Zoom, a videoconferencing platform used by some health providers for telemedicine, since the pandemic [26] highlight the need to assess and address data practices of digital platforms before using them for sensitive purposes such as the provision of health care.

In effect, after this rule change, a large portion of health personal information that is communicated through or stored on digital tools for mental health care is now not covered by HIPAA. HIPAA protections apply to “covered entities” including health care providers and institutions but not to telehealth technologies including apps that collect information directly from consumers [27]. Given the major shift toward the use of telehealth and apps for mental health care, an increasing portion of sensitive mental health information is now shared and stored by technologies that do not provide protections for that information. Even nonhealth digital data collected on a smartphone, such as location, can be used to generate highly personal behavioral and health information such as evidence of depressive or manic episodes, psychosis, or onset of Parkinson Disease [28-30]. Developers of mental health apps have been shown to engage in misleading privacy and data practices, sharing users’ personal data with third parties without their consent [31]. Behavioral health information is a valuable commodity, and it is likely that companies will take further advantage of the lax security and privacy landscape [32]. The potentially unchecked collection of user data through consumer mental health technologies, allowing for personal information to connect individual users to mental health concerns (eg, heightened anxiety for fear of infection), could especially violate a user’s right to privacy. For example, people could be targeted for advertisements that take advantage of their anxieties around COVID-19 by drawing behavioral inferences from their personal information [33]. At a time when the need for trust in telehealth and mental health apps has intensified, it is important to ensure accountability and mechanisms to mitigate unauthorized or unpredictable use of mental health data.

Appropriate informed consent regarding data practices are often invoked as part of ethical approaches to privacy and data protection for digital mental health in psychiatric and clinical care. It is therefore critical to note that the changes to HIPAA, in effect, mean that patients, as consumers of the technologies used for telehealth, are responsible for availing themselves of the consumer technology company’s privacy policy [34]. In the consumer domain, data protection and privacy information are generally presented through dense legal terms and conditions that are difficult for users to parse. Improvements to consent and *terms and conditions* regarding privacy and data use are needed. The General Data Protection Regulation in Europe and California’s Consumer Privacy Act provide some models for giving individuals clearer information regarding personal data use and options to consent to certain uses [35,36].

Yet, the HIPAA rule change raises broader questions regarding whether it is appropriate to place the burden on patients and consumers to understand and respond to how technologies are using their mental health data. Expecting patients, particularly those who may have pressing mental health needs, to understand and weigh issues, such as additional health inferences drawn from their data, the third parties to whom the data may be sold, and the various implications and repercussions of having their data sold, is unreasonable. In the consumer context, the lack of transparency in privacy policies has sometimes been justified by the argument that the use of these technologies is usually not necessary for the health and well-being of the user. However, this is not the case in the clinical context. If patients have no choice but to use the consumer technologies to obtain health care, then they have no choice but to acquiesce to the exploitative privacy practices of technology companies. In addition to focusing on consent and transparency as ways to address deficiencies in data protection, regulations to limit how data may be used for targeted marketing, actions by the Federal Trade Commission to address unfair or deceptive practices, and additional regulations in lieu of HIPAA may be needed to protect patients.

Data collected by digital technology and social media can be valuable for research into the mental health impact of COVID-19 and for developing evidence-based interventions [37]. HIPAA already contains provisions that allow for covered entities to convey personal health information to relevant health authorities for public health purposes. Even when such research is performed outside of HIPAA-covered entities, there is just as much of an imperative to protect private health information. Neglecting to put strong regulations in place now might risk a *new normal* of lowered privacy standards in the future. Although the context of a pandemic justifies sharing of confidential information for goals such as contact tracing, the practice becomes more problematic when the threat to society is less urgent. To avoid inappropriate government or commercial tracking of data, regulations and guidelines must be developed to address tracking of personal data for health research purposes, clearly identifying the types of data that may be tracked, the length of time such data may be stored, and the entities that may access this data [38].

## Safety and Accountability

Increased access to digital mental health tools, when paired with appropriate clinical oversight, can improve and expand care, especially to underserved areas. At the same time, the large-scale reliance on digital tools during the pandemic has underscored and exacerbated the existing gaps in accountability and oversight in digital mental health. There are not yet broadly accepted ethical guidelines for the provision of digital mental health care. Digital technology can have disparate risks and benefits for research and treatment in different populations. For example, although some apps and digital tools have demonstrated benefit for people with moderate anxiety or depression, there is an urgent need to determine best practices and digital tools for providing socially distanced care for people with more severe mental illness [39]. Furthermore, the majority of consumer apps are not evidence-based and some even contain harmful content [40].

The population most likely to use digital mental health apps are people with pre-existing or newly diagnosed mental health problems such as anxiety or depression. Mental health issues add specific layers of vulnerability to a person and may compound and exacerbate coexisting psychosocial crises. If a person in severe emotional crisis, for example, due to depression, is already in a financially precarious position and then loses their job (and health insurance) because of the pandemic, they might be prone to substituting (costly) quality in-person mental health care with a cheaper, yet often unvetted, mental health app. In such a situation, the compounding effects of pathogenic (depression) and socioeconomic (joblessness) vulnerabilities might both interfere with their capacity to evaluate or use a mental health app appropriately or make an informed decision regarding the protection of their mental privacy.

In the United States, the Food and Drug Administration (FDA) relaxed regulation of mental health apps for depression, anxiety, and insomnia to facilitate expanded use of digital health tools during the pandemic [41-43]. Lowering standards may lead to efficiency in the short-term, but the widespread adoption of low-quality technology during the pandemic could lead to a long-term substandard tier of service. One specific concern is that the change in FDA oversight for digital mental health tools further opens the gate for unvetted apps and other services that are put on the market purely for profiteering off of the ongoing mental health crisis rather than providing actual relief for patients. The lack of clear regulatory oversight and guidance during the pandemic will also make it difficult to hold developers and companies liable and responsible for potential harms a user may sustain from interacting with insufficiently vetted digital mental health. Research to identify the benefits and burdens of digital mental health tools, as well as best practices in their application, is urgently needed. Therefore, we emphasize the need for the FDA or other centralized body to coordinate the systematic gathering of data on possible adverse effects of digital mental health tools. This could include establishing a secure channel for user feedback and other means of research.

With the shift to telehealth, many mental health practitioners found themselves encountering new terrain with insufficient guidance or training. Difficulties include the need for more careful safety planning for patients who are at high risk, maintaining professional boundaries in the newly informal virtual space, and designing the physical space to both frame the patient encounter and maintain work-life balance for the therapist [44]. The use of video in teleconference presents challenging questions regarding managing what a client might see of one’s home environment and vice versa. Therapists also need to consider how and when to handle situations where a client might not be alone for confidential one-on-one therapy. There are anecdotal observations supporting some advantages to telehealth for mental health care, such as clients being more likely to show up for an online therapy session [45,46]. However, it will be important to track these gains to see if they are retained after the pandemic and as patients adjust their expectations for care.

Even before the pandemic, many mental health professionals had concerns regarding their legal liability when using digital mental health tools, particularly when it came to assessment and treatment of individuals with severe mental illness or high suicide risk [47]. In the wake of the pandemic, there is added tension for professionals as they balance the need to ensure the continued care for their clients with caution regarding additional liability from their use of digital tools. Physicians are accountable through the law of tort and under the regulatory licensing systems of the jurisdiction in which they provide care [48]. The standard of “reasonable care” creates a duty to exercise a level of care, skill, and judgment that falls within local professional norms. The relative novelty of many digital health tools means there are not yet established standards that courts can use to determine when clinicians fail to meet their duty to patients. There are unclear legal risks involved in cases where a professional relies on a mental health app in an unsuitable case nor is it established how much due diligence a professional is expected to do into the quality and functioning of mental health apps before adoption of them [49]. This is complicated by the context of a pandemic in which clinicians have had to shift quickly to digital tools without the opportunity for training or gradual implementation. Furthermore, not all malpractice insurance providers cover claims arising from telehealth services [50].

In addition to the provision of reasonable care, physicians are expected to obtain informed consent to treatment and to safeguard the confidentiality of medical information. These legal responsibilities can be challenging in relation to poorly understood apps with opaque data management and the potentially large volume of incoming information from digital mental health tools. Given the current need to use digital mental health tools to provide mental health care during the pandemic, the lacuna of norms and regulations leaves providers and patients without clear guidance regarding accountability. Local state-based licensing boards, which serve an oversight function for physician practice, will have a difficult time managing the national and global nature of digital health. Professional organizations such as the American Psychiatric Association (APA) and the American Academy of Clinical Psychology play an important role in establishing guidelines and standards of care for practitioners in these new circumstances. The development of consistent policies around the use of digital mental health tools is vital to ensuring clinicians are not discouraged from using new therapies out of fear of litigation.

Even after the pandemic, we may see a rise in mental health app use if it becomes normalized during the pandemic. If insurers similarly shift to encouraging mental health apps as a replacement for in-person services due to cost, it will be vital that apps are only reimbursed if they provide effective care. Germany’s Digital Health Act, intended to accelerate the use of digital health tools during the pandemic, provides a model for navigating these concerns by requiring companies to submit evidence of safety and efficacy before they are allowed to receive reimbursement [51,52]. Similar regulation could help to provide a more consistent system for evaluation of digital health tools and ensure that users have access to safe products.

Although the pandemic has increased the number of people turning to apps to address mental health issues, there remains a need for a clear framework to allow users to understand the risks and benefits of selecting a digital mental health tool from the sea of options. The APA’s database evaluating mental health apps is a step in the right direction but is geared toward clinicians rather than consumers [53]. In contrast, Australia has a consumer-facing online government portal that provides evidence-based information regarding mental health apps developed by universities, government, and public health agencies [54]. The issue of vulnerability in relation to mental health might be a useful lens to better understand what the optimal balance could be between the benefits of continual access to therapy and the associated possible trade-offs such as involuntary exposure or violation of a patient’s privacy (eg, regarding their home environment). Guidance for therapists will need to be informed by further research and include frameworks for evaluating and handling the trade-offs that may be presented when conducting digital mental health therapy. The context of the pandemic forced much of therapy online, but a true revolution in digital mental health will require evidence-based interventions that are more than simply traditional practice in a virtual space.

## Access and Fairness

Even as the social and psychological burdens of the pandemic are expected to increase rates of mental health issues [55,56], the economic fallout of the pandemic is leading to further slashing of already underresourced mental health budgets [57,58]. The impact of COVID-19 has laid bare systemic health inequities and exerted a disproportionate impact on vulnerable populations such as older adults, racial and ethnic minorities, people living with disabilities, and people who are homeless [59,60]. Although a majority of people using telehealth have reported satisfaction [61], there are indications that telehealth has not served Black Americans or Latinx populations as well. Community mental health centers, which disproportionately serve Black and Latinx people, are much less likely to be prepared to implement digital mental health technologies [62]. Black and Latinx patients are less likely than White patients to use online health services and more likely to express reservations regarding privacy and suspicion regarding the quality of telehealth [63]. The conditions of the pandemic underscore the need to prioritize approaches that can provide improved care for groups that have repeatedly seen the least benefits from the mental health system and emerging technologies.

Since the pandemic, there has been renewed focus on how the design of health technologies may unfortunately reflect and reinforce existing biases and health disparities [64]. As with other areas of health research and technology, non-White populations are likely to be underrepresented in the data used to develop digital mental health algorithms and tools [65]. Informational and treatment apps also may not be tailored appropriately for people of different racial, linguistic, ethnic, or cultural backgrounds. Even with digital tools that are simply meant to connect patients with mental health providers, the lack of racial and cultural diversity in the pool of clinicians and therapists can create barriers to mental health care [66]. In the United States, allowing mobility of professional licensure could help create more diverse care teams and improve access to clinicians who speak the same language as patients.

Furthermore, digital mental health tools require proper training and oversight to use effectively. Insufficient resources for adequate training of mental health professionals serving low-income demographics may mean that quality mental health care will still not be accessible despite expansion of digital mental health [67,68]. It is vital for developers, researchers, and clinicians to address potential areas of bias and plan for how to engage culturally diverse populations as well as vulnerable populations. Given that low-income groups may face worse health outcomes from contracting COVID-19 and greater economic uncertainty after the pandemic, they can least afford this result.

In the United States, where there is a patchwork of public and private health care options for mental health care, reimbursement mechanisms are a key issue for fair access. During the pandemic, reimbursement for telehealth has expanded due to social distancing requirements, but postpandemic, reimbursement for digital tools may be lower than for traditional approaches. Vulnerable populations offered mental health access during the pandemic may only have them taken away once the main crisis has ended. Inadequate evaluation mechanisms for digital health tools can also make it difficult to identify and reimburse the tools that provide quality mental health care.

As with other digital tools for health-related applications, it is important to ensure fair access, particularly to vulnerable user groups. At the same time, low-access barriers for end users often goes along with low priorities for the security and data privacy of digital tools. With the continuing crisis of mental health care provision during the pandemic, users might trade off privacy and access in ways that are not commensurate with accepted norms of human dignity and fairness. One pathway to take off pressure from individuals in dire need of digital mental health provision could be to prioritize the development and dissemination of FDA-vetted tools that conform to high standards of psychiatric care and scientific evidence. It will also be imperative to invest resources in improving the digital tools available through community mental health centers and provide for training and outreach programs for digital mental health tools in underresourced communities.

## Conclusion

The COVID-19 pandemic has forced rapid adoption of digital mental health tools. However, the ethical challenges regarding privacy, fairness, transparency, and accountability remain unresolved. There is a pressing need for interdisciplinary, coordinated research efforts to understand the effects of this large-scale shift to digital mental health tools. Multidisciplinary efforts should also incorporate the input of people with lived experience of mental health issues [69]. Policy makers must assess whether to adopt new regulations to protect privacy and ensure transparency or whether modification of existing standards will be sufficient. Policy must be developed in conjunction with service users to avoid creating new inequalities in access to mental health [70]. Ultimately, the current crisis may be an opportunity to unpack the great potential for digital mental health tools to improve public health.

## Abbreviations

APA: American Psychiatric Association
FDA: Food and Drug Administration
HIPAA: Health Information Portability and Accountability Act

## References

[1] Gao J, Zheng P, Jia Y, Chen H, Mao Y, Chen S, Wang Y, Fu H, Dai J (2020). Mental health problems and social media exposure during COVID-19 outbreak. PLoS One.

[2] Qiu J, Shen B, Zhao M, Wang Z, Xie B, Xu Y (2020). A nationwide survey of psychological distress among Chinese people in the COVID-19 epidemic: implications and policy recommendations. Gen Psychiatr.

[3] Tayag Y (2020). Who’s most at risk in the Covid-19 mental health crisis. Medium Coronavirus Blog.

[4] Hawkley LC, Capitanio JP (2015). Perceived social isolation, evolutionary fitness and health outcomes: a lifespan approach. Philos Trans R Soc Lond B Biol Sci.

[5] Armitage R, Nellums LB (2020). COVID-19 and the consequences of isolating the elderly. Lancet Public Health.

[6] Usher K, Bhullar N, Durkin J, Gyamfi N, Jackson D (2020). Family violence and COVID-19: increased vulnerability and reduced options for support. Int J Ment Health Nurs.

[7] (2020). Double jeopardy: COVID-19 and behavioral health disparities for Black and Latino communities in the U.S. (submitted by OBHE). Substance Abuse and Mental Health Services Administration.

[8] Lee J (2020). Mental health effects of school closures during COVID-19. Lancet Child Adolesc Health.

[9] Jee C (2020). Many covid-19 survivors will be left traumatized by their ICU experience. MIT Technology Review.

[10] Hatch R, Young D, Barber V, Griffiths J, Harrison DA, Watkinson P (2018). Anxiety, depression and post traumatic stress disorder after critical illness: a UK-wide prospective cohort study. Crit Care.

[11] Gold J (2020). The hidden Covid-19 crisis: health care workers' mental health. STAT.

[12] Vindegaard N, Benros ME (2020). COVID-19 pandemic and mental health consequences: systematic review of the current evidence. Brain Behav Immun.

[13] Zacher H, Rudolph CW (2020). Individual differences and changes in subjective wellbeing during the early stages of the COVID-19 pandemic. Am Psychol.

[14] Holman EA, Thompson RR, Garfin DR, Silver RC (2020). The unfolding COVID-19 pandemic: a probability-based, nationally representative study of mental health in the United States. Sci Adv.

[15] Torous J, Jän Myrick K, Rauseo-Ricupero N, Firth J (2020). Digital mental health and COVID-19: using technology today to accelerate the curve on access and quality tomorrow. JMIR Ment Health.

[16] Figueroa CA, Aguilera A (2020). The need for a mental health technology revolution in the COVID-19 pandemic. Front Psychiatry.

[17] Moreno C, Wykes T, Galderisi S, Nordentoft M, Crossley N, Jones N, Cannon M, Correll CU, Byrne L, Carr S, Chen EYH, Gorwood P, Johnson S, Kärkkäinen H, Krystal JH, Lee J, Lieberman J, López-Jaramillo C, Männikkö M, Phillips MR, Uchida H, Vieta E, Vita A, Arango C (2020). How mental health care should change as a consequence of the COVID-19 pandemic. Lancet Psychiatry.

[18] Torous J, Keshavan M, Gutheil T (2014). Promise and perils of digital psychiatry. Asian J Psychiatr.

[19] Fulton D (2020). FDA eases entry for psychiatry apps during COVID-19 crisis. Regulatory Affairs Professionals Society.

[20] Emerging Issues Task Force‚ International Neuroethics Society (2019). Neuroethics at 15: the current and future environment for neuroethics. AJOB Neurosci.

[21] Martinez-Martin N, Kreitmair K (2018). Ethical issues for direct-to-consumer digital psychotherapy apps: addressing accountability, data protection, and consent. JMIR Ment Health.

[22] Adam EK, Chyu L, Hoyt LT, Doane LD, Boisjoly J, Duncan GJ, Chase-Lansdale PL, McDade TW (2011). Adverse adolescent relationship histories and young adult health: cumulative effects of loneliness, low parental support, relationship instability, intimate partner violence, and loss. J Adolesc Health.

[23] Aitken M, de St Jorre J, Pagliari C, Jepson R, Cunningham-Burley S (2016). Public responses to the sharing and linkage of health data for research purposes: a systematic review and thematic synthesis of qualitative studies. BMC Med Ethics.

[24] (2020). Notification of enforcement discretion for telehealth remote communications during the COVID-19 nationwide public health emergency. HHS.gov.

[25] (2020). FAQs on telehealth and HIPAA during the COVID-19 nationwide public health emergency. HHS.gov.

[26] Chen BX (2020). The lesson we are learning from Zoom. The New York Times.

[27] 45 CFR § 164.306 security standards: general rules. govinfo.

[28] Saeb S, Zhang M, Karr CJ, Schueller SM, Corden ME, Kording KP, Mohr DC (2015). Mobile phone sensor correlates of depressive symptom severity in daily-life behavior: an exploratory study. J Med Internet Res.

[29] Erdogdu Sakar B, Serbes G, Sakar CO (2017). Analyzing the effectiveness of vocal features in early telediagnosis of Parkinson's disease. PLoS One.

[30] Wachter S, Mittelstadt B (2019). A right to reasonable inferences: re-thinking data protection law in the age of big data and AI. Columbia Business Law Rev.

[31] Huckvale K, Torous J, Larsen ME (2019). Assessment of the data sharing and privacy practices of smartphone apps for depression and smoking cessation. JAMA Netw Open.

[32] Chen A (2019). Why it's time to rethink the laws that keep our health data private. The Verge.

[33] McCoy MS, Libert T, Friedman AB (2020). Online privacy loss: another Covid-19 aftershock. STAT.

[34] Bassan S (2020). Data privacy considerations for telehealth consumers amid COVID-19. J Law Biosciences.

[35] (2016). Regulation (EU) 2016/679 of the European Parliament and of the Council of 27 April 2016 on the protection of natural persons with regard to the processing of personal data and on the free movement of such data, and repealing Directive 95/46/EC (General Data Protection Regulation). EUR-Lex.

[36] California Consumer Privacy Act of 2018. California Legislative Information.

[37] Lynch S (2020). What Twitter reveals about COVID-19’s impact on our mental health. Standard University Human-Centered Artificial Intelligence.

[38] Mello MM, Wang CJ (2020). Ethics and governance for digital disease surveillance. Science.

[39] Torous J, Keshavan M (2020). COVID-19, mobile health and serious mental illness. Schizophr Res.

[40] Larsen ME, Huckvale K, Nicholas J, Torous J, Birrell L, Li E, Reda B (2019). Using science to sell apps: evaluation of mental health app store quality claims. NPJ Digit Med.

[41] (2020). Enforcement policy for digital health devices for treating psychiatric disorders during the coronavirus disease 2019 (COVID-19) public health emergency. US Food and Drug Administration.

[42] Brauser D (2020). COVID-19: dramatic changes to telepsychiatry rules and regs. Medscape.

[43] Al-Faruque F (2020). FDA relaxes regs for COVID-19 mental health apps. Medtech Insight.

[44] Sasangohar F, Bradshaw MR, Carlson MM, Flack JN, Fowler JC, Freeland D, Head J, Marder K, Orme W, Weinstein B, Kolman JM, Kash B, Madan A (2020). Adapting an outpatient psychiatric clinic to telehealth during the COVID-19 pandemic: a practice perspective. J Med Internet Res.

[45] Kopelovich SL, Monroe-DeVita M, Buck BE, Brenner C, Moser L, Jarskog LF, Harker S, Chwastiak LA (2020). Community mental health care delivery during the COVID-19 pandemic: practical strategies for improving care for people with serious mental illness. Community Ment Health J.

[46] Uscher-Pines L, Sousa J, Raja P, Mehrotra A, Barnett ML, Huskamp HA (2020). Suddenly becoming a "Virtual Doctor": experiences of psychiatrists transitioning to telemedicine during the COVID-19 pandemic. Psychiatr Serv.

[47] Groth T, Boccio DE (2019). Psychologists' willingness to provide services to individuals at risk of suicide. Suicide Life Threat Behav.

[48] Cortez N (2018). The evolving law and ethics of digital health. Digital Health: Scaling Healthcare to the World.

[49] Terry NP, Wiley LF (2016). Liability for mobile health and wearable technologies. Ann Health Law.

[50] Hoffman D (2020). Increasing access to care: telehealth during COVID-19. J Law Biosci.

[51] Matthies VH (2019). DVG – a summary of Germany’s new law for digital health applications. Health Innovation Hub.

[52] Gerke S, Stern AD, Minssen T (2020). Germany's digital health reforms in the COVID-19 era: lessons and opportunities for other countries. NPJ Digit Med.

[53] App advisor. American Psychiatric Association.

[54] Head to Health.

[55] Qiu J, Shen B, Zhao M, Wang Z, Xie B, Xu Y (2020). A nationwide survey of psychological distress among Chinese people in the COVID-19 epidemic: implications and policy recommendations. Gen Psychiatr.

[56] Vindegaard N, Benros ME (2020). COVID-19 pandemic and mental health consequences: systematic review of the current evidence. Brain Behav Immun.

[57] (2018). Mental Health Atlas 2017. World Health Organization.

[58] Kaiser Health News (2020). Colorado, like other states, trims mental health programs amid health crisis. The Denver Post.

[59] Webb Hooper M, Nápoles AM, Pérez-Stable EJ (2020). COVID-19 and racial/ethnic disparities. JAMA.

[60] van Deursen AJ (2020). Digital inequality during a pandemic: quantitative study of differences in COVID-19-related internet uses and outcomes among the general population. J Med Internet Res.

[61] Bestsennyy O, Gilbert G, Harris A, Rost J (2020). Telehealth: a quarter-trillion-dollar post-COVID-19 reality?. McKinsey and Company.

[62] George S, Hamilton A, Baker R (2012). How do low-income urban African Americans and Latinos feel about telemedicine? A diffusion of innovation analysis. Int J Telemed Appl.

[63] Gray DM, Joseph JJ, Olayiwola JN (2020). Strategies for digital care of vulnerable patients in a COVID-19 world—keeping in touch. JAMA Health Forum.

[64] Gianfrancesco MA, Tamang S, Yazdany J, Schmajuk G (2018). Potential biases in machine learning algorithms using electronic health record data. JAMA Intern Med.

[65] Bowens S (2020). Health watch: closing the gap when it comes to mental health care for minorities. Wilmington Star-News.

[66] Chen I, Szolovits P, Ghassemi M (2019). Can AI help reduce disparities in general medical and mental health care?. AMA J Ethics.

[67] Conrad R, Rayala H, Diamond R, Busch B, Kramer N (2020). Expanding telemental health in response to the COVID-19 pandemic. Psychiatric Times.

[68] Velasquez D, Mehrotra A (2020). Ensuring the growth of telehealth during COVID-19 does not exacerbate disparities in care. Health Affairs.

[69] Holmes EA, O'Connor RC, Perry VH, Tracey I, Wessely S, Arseneault L, Ballard C, Christensen H, Cohen Silver R, Everall I, Ford T, John A, Kabir T, King K, Madan I, Michie S, Przybylski AK, Shafran R, Sweeney A, Worthman CM, Yardley L, Cowan K, Cope C, Hotopf M, Bullmore E (2020). Multidisciplinary research priorities for the COVID-19 pandemic: a call for action for mental health science. Lancet Psychiatry.

[70] Illes J, Bird SJ (2006). Neuroethics: a modern context for ethics in neuroscience. Trends Neurosci.

